# Nonhuman Primate Models of Chikungunya Virus Infection and Disease (CHIKV NHP Model)

**DOI:** 10.3390/pathogens4030662

**Published:** 2015-09-16

**Authors:** Rebecca Broeckel, Nicole Haese, Ilhem Messaoudi, Daniel N. Streblow

**Affiliations:** 1 Vaccine & Gene Therapy Institute, Oregon Health & Science University, 505 NW 185th Ave, Beaverton, OR 97006, USA; E-Mails: broecker@ohsu.edu (R.B.); haese@ohsu.edu (N.H.); 2 Division of Biomedical Sciences, School of Medicine, University of California-Riverside, Riverside, CA 92521, USA; E-Mail: ilhem.messaoudi@ucr.edu

**Keywords:** chikungunya virus, nonhuman primate, polyarthritis, pathogenesis, immunity

## Abstract

Chikungunya virus (CHIKV) is a positive-sense RNA virus transmitted by *Aedes* mosquitoes. CHIKV is a reemerging Alphavirus that causes acute febrile illness and severe and debilitating polyarthralgia of the peripheral joints. Huge epidemics and the rapid spread of CHIKV seen in India and the Indian Ocean region established CHIKV as a global health concern. This concern was further solidified by the recent incursion of the virus into the Western hemisphere, a region without pre-existing immunity. Nonhuman primates (NHPs) serve as excellent animal models for understanding CHIKV pathogenesis and pre-clinical assessment of vaccines and therapeutics. NHPs present advantages over rodent models because they are a natural amplification host for CHIKV and they share significant genetic and physiological homology with humans. CHIKV infection in NHPs results in acute fever, rash, viremia and production of type I interferon. NHPs develop CHIKV-specific B and T-cells, generating neutralizing antibodies and CHIKV-specific CD4^+^ and CD8^+^ T-cells. CHIKV establishes a persistent infection in NHPs, particularly in cynomolgus macaques, because infectious virus could be recovered from spleen, liver, and muscle as late as 44 days post infection. NHPs are valuable models that are useful in preclinical testing of vaccines and therapeutics and uncovering the details of CHIKV pathogenesis.

## 1. Introduction

Chikungunya virus (CHIKV) is a mosquito-transmitted RNA virus that causes acute febrile illness and severe debilitating joint pain. CHIKV belongs to the Semliki Forest virus clade of Old World Alphaviruses [1,2]. CHIKV historically caused sporadic outbreaks in Africa and Southeast Asia [3]. Globalization and ease of overseas travel have increased risk of spread of CHIKV to other regions without preexisting immunity and competent *Aedes* mosquito vectors. In densely populated regions of susceptible humans it is estimated that the infection rate is between 30%–75% [4,5,6]. The 2004–2007, endemic in the Indian Ocean region and India demonstrated the potential of CHIKV to rapidly spread and establish itself in new geographical regions. In December of 2013, CHIKV achieved autochthonous transmission in the Western hemisphere in the Caribbean Islands [7,8] and subsequently became established in Central and South America, Mexico, and the mainland United States (Florida) [9]. According to the Centers for Disease Control and Prevention (CDC), over 1.5 million cases have occurred in the Western hemisphere [10]. Risk of spread could be reduced with increased vector control such as removal of mosquito breeding sites, mosquito repellent, and community awareness.

CHIKV has a 12 kb positive-sense single stranded RNA genome consisting of four nonstructural proteins (nsp1-4) and five structural proteins (capsid, E3, E2, 6k, and E1) [11]. CHIKV is an enveloped virus studded with 80 trimers of E1/E2 dimers [12]. While the cellular receptor for the virus is unknown, CHIKV enters cells via clathrin-mediated endocytosis and the capsid containing genomic RNA is released into the cytoplasm [13]. The nonstructural proteins are translated from the genomic RNA and together form the NSP protein complex that includes the viral RNA-dependent RNA polymerase which is necessary for minus and plus strand genomic RNA replication [14]. The NSP complex also synthesizes the subgenomic RNA from minus-strand RNA that codes for the structural polyprotein. During translation of the structural polyprotein, the capsid autocatalytically cleaves itself, exposing an N-terminal ER localization signal [15,16]. The envelope proteins are folded in the endoplasmic reticulum (ER), and they undergo further processing and glycosylation in the ER and Golgi secretory network before being presented on the plasma membrane. The capsid complex assembles around the genomic RNA and interacts with the envelope proteins at the plasma membrane [17,18]. The virions bud and are released for the next round of infection.

The acute stage of CHIKV disease, in humans, is characterized by rash, high fever, headache, and arthralgia. These symptoms can appear within 3–7 days and typically last for two weeks. After a bite from an infected *Aedes* mosquito, the virus replicates locally in the skin and disseminates via the blood to peripheral joints, muscle, tendons, liver, and lymph nodes. Replication in these tissues results in inflammation, synovitis, and tenosynovitis causing intense pain in the joints, tendons, and muscles. The sub-acute phase of CHIKV or convalescent phase is associated with resolution of viremia, fever, and induction of CHIKV-specific T-cell and antibody immune responses. A significant portion of those infected will enter into the chronic phase of CHIKV disease, characterized by recurring polyarthritis, myalgia, and tenosynovitis [19,20], that can last for months to years post infection [19,21,22,23,24,25]. The prevailing hypothesis is that incomplete clearance of CHIKV from specific tissues leads to chronic infection. Persistent CHIKV may replicate at low levels in the joints and muscles, causing long-term arthritic pain and myalgia. Risk factors in developing persistent CHIKV associated rheumatic manifestations are increased age (>45 years old), preexisting arthritis or joint disease, hypertension, and increased disease severity during the acute phase of disease [24]. Though considered a relatively benign disease, CHIKV can cause serious, even fatal disease in neonates, aged, and immunocompromised populations. Other underlying medical conditions such as diabetes, cardiovascular disease, neurological disorders, and chronic pulmonary diseases are risk factors for developing severe CHIKV disease [26]. Atypical symptoms include encephalitis, seizures, heart failure and arrhythmias, renal failure, skin lesions, and blindness [26,27,28]. There is documented evidence of perinatal mother-to-child transmission resulting in severe disease, including fever, rash, hemorrhagic disease, and seizures from La Reunion, an Indian Ocean Island where 30%–40% of the population was infected during the 2005–2006 Indian Ocean Island epidemic [29].

CHIKV has natural cycles of reemergence that occur in patterns of several years to decades after previous epidemics [3,30]; the virus is thought to maintain itself in NHPs or other vertebrate reservoirs and is reintroduced to regions with susceptible populations lacking preexisting immunity to CHIKV. The transmission cycle from NHPs (or other vertebrate such as rodents) to humans using a mosquito vector is known as the zoonotic sylvatic transmission cycle. However, CHIKV does not require an animal intermediate. In densely populated areas CHIKV utilizes an urban transmission cycle involving only humans and mosquitoes. The most important mosquito vectors in urban transmission cycles are the anthropophilic species *Aedes aegypti* and *Aedes albopictus*. The zoonotic sylvatic transmission cycle is believed to occur in West and Central Africa involving forest-dwelling *Aedes* mosquitoes, NHPs, and other vertebrate reservoirs [31,32,33,34]. It is possible that a zoonotic sylvatic transmission cycle exists in Asia because of isolation of infectious virus from NHPs in Malaysia [35] and CHIKV-specific antibodies present in Asian NHPs [36,37]; however, the nature of the transmission cycle in Asia needs to be more thoroughly studied. Both humans and NHPs serve as amplification hosts for CHIKV, thus highlighting the importance of studying CHIKV in NHPs. Genetic analysis of several CHIKV isolates revealed that there are three distinct CHIKV lineages: the Asian clade, West African clade, and East/Central/South African (ECSA) clade [38]. Sequence analysis showed that CHIKV isolates from the Indian Ocean outbreak were of the ECSA lineage. Importantly, CHIKV isolates from the Indian Ocean outbreak contained a point mutation in E1 (A226V) that increased its infectivity in *Aedes albopictus* mosquitoes [39,40,41]. The CHIKV-LR isolates were sampled during the Indian Ocean outbreak and contain this point mutation. Other CHIKV isolates commonly used in NHP studies include the Senegal isolate CHIKV-37997 from the West African clade and the ECSA isolate CHIKV-DHS-4263 obtained from a traveler in India during the Indian Ocean Island outbreak [42]. The circulating strain in the Caribbean stems from the Asian lineage, but the Caribbean isolates have not been used in any reported NHP studies [43].

## 2. Animal Models of CHIKV

### 2.1. Mouse Models of CHIKV Infection and Disease

There are many different animal models that have been used for studying CHIKV pathogenesis, mostly involving different laboratory mouse strains. Of note, neonatal mice and mice that lack the type I interferon receptor are highly susceptible to CHIKV infection, indicating the importance of type I interferon in controlling virus infection [44]. Rag1^−/−^ mice, which are deficient in mature B and T-cells, develop a persistent CHIKV infection in which infectious virus can be recovered from tissues and serum for the lifetime of the mouse [45,46]. Transfer of immune sera into the Rag1^−/−^ mice results in a transient reduction of infectious virus in the serum [45] that returns after a few weeks [46]. This demonstrates that though antibodies can clear virus in the serum for a short time, they are not sufficient for complete viral clearance. The direct role for T-cells still remain elusive, but it has been shown that CD4^+^ T-cells may play a role in joint swelling [47]. Infection of young and adult C57BL/6 mice with CHIKV in the footpad can recapitulate the arthritis and myositis symptoms seen in humans [48,49]. In addition, CHIKV RNA can be detected in this mouse model for several weeks post infection, providing evidence for CHIKV persistence [45,48]. However, unlike the polyarthritis that occurs during human CHIKV infections, arthritis in mice is usually limited to the infected ipsilateral joint. There are several mouse models available for understanding different aspects of CHIKV pathogenesis and immunity; however, uncovering the details of CHIKV infection in NHPs may be more relevant to human infection and disease due to immunological and physiological similarities. Thus, the NHP model of CHIKV infection more accurately predicts the human outcomes of antiviral therapeutics and vaccines.

### 2.2. NHP Models of CHIKV Infection

The first CHIKV NHP experiments were performed in 1953 in the Newala district of Tanzania. R.W. Ross demonstrated that rhesus macaques developed neutralizing antibodies to inoculations with viremic human sera [50]. Other early studies in rhesus macaques showed development of viremia 2–4 days post infection (dpi), production of neutralizing antibodies, and protection from reinfection [51]. Three recent studies have provided a more detailed picture of CHIKV pathogenesis using the following animal models: adult (6–13 years old) and aged rhesus macaques (>17 years old) [52]; cynomolgus macaques [53]; and pregnant rhesus macaques [42].

#### 2.2.1. Acute Stage of CHKV Infection in NHP

The acute stage of CHIKV infection in NHPs has similarities to acute CHIKV infection in humans. In humans, viremia lasts an average of 4–6 days, but can last up to 12 days post-onset of illness, with viremia reaching between 10^5^ to 10^12^ pfu/mL [54,55,56,57,58]. Development of viremia in CHIKV-infected NHPs has been studied since the 1960s [42,51,53,59,60,61]. CHIKV has been experimentally administered to NHP by a variety of means including subcutaneous, intravenous, and intramuscular injections. However, it is unclear how the specific routes of infection and reliance on mosquito-specific delivery mechanisms and/or saliva effect CHIKV transmission and subsequently pathogenesis. Elegant intravital microscopy analysis of feeding patterns of Anopheles mosquitoes demonstrated the impact of mosquito age and malaria infection on increasing feeding behavior and pathogen transmission, which highlights the importance of determining pathogen: vector interactions and their role in mosquito transmission [62]. In the early CHIKV macaque studies, monkeys infected with either CHIKV-African E-103 or CHIKV-Asian BAH-306 developed viremia that lasted 4-5 days with peak viremia levels occurring 2–4 dpi, reaching 10^4^–10^6^ pfu/mL [51]. Results from more recent studies utilizing rhesus macaques inoculated with either CHIKV-LR or CHIKV-37997 at varying doses (10^7^, 10^8^, or 10^9^ pfu) reported that regardless of inoculating dose, animals were viremic by 2 dpi with resolution around 5 dpi. Animals infected with CHIKV-LR had slightly higher peak viremias compared to CHIKV-37997 infected animals (5.7 × 10^6^ *vs*. 5.9 × 10^5^ vRNA/10 μL plasma, respectively) [59]. Cynomolgus macaques infected with CHIKV-LR at the same dose range also developed peak viremia by 2 dpi with higher viral loads than those observed in rhesus (5 × 10^9^ vRNA/mL plasma) [53]. In contrast to rhesus, the higher the inoculation dose the sooner viremia peaked and the higher peak viremia levels occurred in cynomolgus macaques. Pregnant rhesus macaques infected with CHIKV-37997 or DHS-4263 developed viremia lasting from 1–5 dpi, peaking at 2–3 dpi. Maximum viral load in pregnant rhesus macaques infected with CHIKV-DHS-4263 was significantly higher compared to infection with CHIKV-37997 [42]. These studies show that infection with specific CHIKV isolates such as CHIKV-LR or CHIKV-DHS-4263 results in higher peak viremia compared to CHIKV-37997, and that cynomolgus macaques are generally more susceptible to development of high titer viremia compared to rhesus macaques.

As described for humans, clinical presentation of acute CHIKV infection in NHPs included high fever at 1–2 dpi that persisted for 2–7 days in cynomolgus, 3–7 days in rhesus, and 17–19 days in pregnant rhesus macaques; fever coincided with rash during the first week of infection [42,53,59]. In cynomolgus macaques, severity of symptoms correlated with CHIKV dose. Those animals receiving a high dose of CHIKV-LR (10^7^–10^8^ pfu) exhibited joint swelling of the wrist and ankle in addition to viremia, fever and rash. A moderate dose (10^2^–10^6^ pfu) caused only viremia, fever and rash, while a low dose (10^1^ pfu) caused no clinical symptoms except for viremia in some animals.

Leukopenia has been described following CHIKV infection of both cynomolgus macaques and pregnant rhesus macaques. Additional alterations in the hemogram included monocytopenia, lymphopenia, granulocytosis, and thromobocytopenia. Changes in blood cell numbers correlated with viremia, with the biggest decrease occurring during peak viremia, 2–4 dpi, followed by levels returning to normal by 10–15 dpi. Both lymphocyte and neutrophil counts were significantly decreased in pregnant rhesus macaques at the time of peak viremia (Table 1) [42]. Similar patterns have also been observed during outbreaks of CHIKV in humans [3,19,42]. In a retrospective study of patients infected during the La Reunion outbreak in 2005, lymphopenia was the most common laboratory abnormality, observed in 79% of patients, followed by moderate thrombocytopenia, which was detected in 43.9% of patients [23]. Results from these NHP studies, along with what has been reported during human infection, support the idea that during CHIKV infection at peak viremia, immune cells are recruited from the blood into the surrounding tissues to control viral loads and reduce dissemination.

#### 2.2.2. CHIKV Tissue Dissemination in NHPs

Dissemination of CHIKV from the site of infection to other tissues throughout the body occurs as early as 2 dpi in infected macaques [53]. Infection of cynomolgus macaques with an intermediate dose of CHIKV-LR (10^2^–10^6^ pfu) resulted in virus dissemination to multiple tissues along with mononuclear cell infiltration in the liver and lymphoid tissues of infected animals. Viral RNA was quantified in tissue lysates from liver, lymph node, liver, joint, muscle, skin, brain, and spinal cord of infected cynomolgus macaques. The viral load in most tissues peaked by 6 dpi but remained detectable for longer periods of time in many tissues including lymphoid organs, liver, synovial and muscular tissues, and CSF [53]. In rhesus macaques at 7 dpi, CHIKV was detected in joints, musculoskeletal tissues, heart, lung, liver, kidney, and lymphoid tissues, demonstrating the ability of CHIKV to disseminate to various tissues in this macaque model [63]. Similar to human infections, persistence of CHIKV in the infected adult rhesus monkeys is much more limited when compared to cynomolgus macaques. However, CHIKV-LR persisted in the spleen of aged rhesus macaques [59]. In the pregnant rhesus macaque model viral RNA was detected in the spleen and lymph node at 21 dpi. Detection of virus in other tissues was dependent on the CHIKV strain used for infection [42].

Infection of cynomolgus macaques with CHIKV resulted in histological abnormalities detected primarily in the liver, lymph nodes, and spleen. In the spleen, an increase of mononuclear cell infiltrates into the red pulp was observed 6–97 dpi [53]. Lymph nodes of infected animals also showed increased infiltration of mononuclear cells, primarily into the cortex, which was associated with enlargement of the follicular region 32–44 dpi. In the liver, there were abnormally high levels of hepatocyte death mainly due to apoptosis, peaking at 6 dpi. At an intermediate dose, CHIKV did not cause any major abnormalities in muscle, synovial samples or central nervous tissues. Using a high dose of CHIKV-LR (≥10^7^ PFU) histological findings were detected starting at 5 dpi and remained detectable until 186 dpi in some tissues [53]. The liver, lymph nodes, and spleen presented with the same histological findings as the intermediate dose of CHIKV, but cell infiltration was detectable to 186 dpi [53]. In addition, there were also regions of muscle fiber isolated at 186 dpi with areas of detectable necrosis, associated with monocyte and macrophage infiltrates [53].

The ability of CHIKV to cause neurological symptoms in humans has been reported since the 1960–1970s [64,65,66,67,68] and also observed during more recent outbreaks [26,69,70,71]. Cynomolgus macaques infected with the high dose of CHIKV-LR exhibited symptoms of meningoencephalitis. Analysis of cerebrospinal fluid (CSF) collected from animals with signs of encephalitis, revealed a large number of mononuclear cells at 4 dpi. Characterization of the individual cell populations in the CSF by flow cytometry revealed that the majority of the cells were lymphocytes (67%) with a small monocytes/macrophages contribution (9%) [53]. Neurological symptoms are most commonly manifested in neonates after mother-to-child transmission. The incidence rate of neurological manifestations in adults is much lower; during the recent La Reunion outbreak only 15%–25% of adults that required hospitalization due to CHIKV infection had neurological symptoms [72]. Recapitulating neurological symptoms of CHIKV infection using a relevant infectious animal model has proven difficult. Mother-to-child CHIKV transmission is a rare event, unless the mother is viremic at or near the time of childbirth, in which case transmission can be as high as 50% [73]. CHIKV was not transmitted to the fetus of pregnant rhesus macaques by transplacental transmission because the fetal tissues and placenta were found to be negative for CHIKV [42]. However, this study was limited to animals at gestation days 121–132. Future pregnant rhesus macaque studies need to investigate vertical CHIKV transmission closer to the end of gestation to determine whether the rhesus macaque model mimics CHIKV vertical transmission observed in humans. Additional studies need to be performed in NHP models in order to understand the neurological manifestations following CHIKV infection.

#### 2.2.3. CHIKV Persistence in NHPs

There is mounting evidence for CHIKV persistence in both humans and rhesus macaques. A proportion of people infected with CHIKV may have recurring arthralgia but it is not known whether CHIKV chronic joint pain is caused by low-level persistent CHIKV replication or whether acute CHIKV replication disrupts an inflammatory equilibrium that causes arthritic pain after infection has resolved. The first evidence of CHIKV persistence in nonhuman primates was shown in adult, immunocompetent cynomolgus macaques infected with CHIKV-LR, and infectious virus was recovered as late as 44 days post infection from spleen, liver, and muscle (Table 1) [53]. The authors also postulated that CD68^+^ macrophages might be reservoirs of persistence in the spleen. Another group showed evidence of CHIKV persistence in the spleen and lymph nodes in pregnant rhesus macaques at 21 days post infection by RT-PCR, but no infectious virus was recovered [42]. In addition, they did not detect CHIKV in the fetal tissues or placenta. Our group detected CHIKV RNA at 35 dpi in the spleen of aged, but not adult rhesus macaques. When aged rhesus macaques were given 10^9^ pfu CHIKV-LR, CHIKV RNA was detected in the spleen 35 dpi; however this was not the case when aged rhesus macaques were given the same dosage of CHIKV-37997 [52], suggesting that CHIKV-LR may be more virulent than CHIKV-37997 in aged macaques. Regardless of the macaque model, the consensus is that CHIKV RNA can be detected long-term in the spleen. In addition, CHIKV infections may be more severe and more persistent in cynomolgus macaques compared to rhesus macaques.

**Table 1:** Stages of Chikungunya virus (CHIKV) infection in non-human primates (NHPs).

	(a) Acute Stage 1-6 dpi	(b) Convalescent Stage 7-34 dpi	(c) Persistent stage >35 dpi	Reference
**Symptoms**	Fever, rash, joint swelling, lymphopenia	Resolution of fever and lymphopenia	Not determined	[42,51,53,59,74]
		Rash and joint swelling resolving		
**Virus Detection**	Virus detectable in the blood, lymph nodes, liver, and spleen	Virus detectable in the liver, lymph nodes, spleen, joints, muscles, and organs	Virus detectable in the liver, spleen, joint synovial tissue, and muscle	[42,51,53,59,61,74,75,76,77]
**Innate Response**	Active monocyte and mDC in the blood	Robust pDC, mDC, & monocytes/macrophages	Not determined	[33,34]	
**Cytokine and Chemokine Production**	Increased IFN-α/β & γ, IL-6, TNF-α, IL-15, IL-1Ra, IL-2, CCL-2, CCL-3, CCL-4, & VEGF	Increased levels of IFN-γ, TNF-α, IL-2, CCL-2, CCL-3, & CCL-4	Not determined	[42,53,59]	
**Virus-Specific Immunity**	Virus-specific immunity is developing	T-Cell Response: Virus-specific CD4^+^ and CD8^+^ (EM and CM)	T-Cell Response: Not determined	[42,51,53,59,61,78]	
		B-Cell Response: Virus-specific IgM & IgG	B-Cell Response: Virus-specific IgG		

(a) During the acute stage of infection (1–6 dpi) CHIKV infection of NHPs results in viremia lasting an average of 4–5 days, fever, rash, and joint swelling. A reduction in numerous cell populations of the blood results in monocytopenia, lymphopenia, granulocytosis, and thromobocytopenia. The innate immune response is also initiated during the acute stage, supported by evidence of an increase in the number of monocytes and mDCs in the peripheral blood. Effectors of the type 1 IFN response are detected early in the acute stage, along with other proinflammatory cytokines/chemokines. (b) The convalescent stage of infection (7–34 dpi) is marked by dissemination of the virus from the blood to other organs of the body including the liver, lymph nodes, spleen, joints, muscles, and organs. The innate immune response continues, and the adaptive immune response is initiated during this stage of infection, including T and B-cell responses, and the production of both IgM and IgG virus specific antibodies. (c) During the persistent stage of infection (>35 dpi) in NHPs, virus remains detectable in the liver, spleen, joint synovial tissues, and muscle. CHIKV is capable of persisting within NHP CD68^+^ macrophages of the spleen. Virus specific IgG antibody responses have also been detected during this stage.

#### 2.2.4. NHP Immune Responses Following CHIKV Infection

##### Innate Immune Cell Activation in NHP

Research by our group reported a difference in both the monocyte and dendritic cell (DC) numbers after CHIKV infection between adult and aged rhesus macaques infected with CHIKV-LR or CHIKV-37997. The levels of plasmacytoid DCs (pDCs) and myeloid DCs (mDCs) increased at 14 dpi before returning to baseline levels following infection of adult rhesus macaques with CHIKV-LR. In contrast, adults infected with CHIKV-37997 had high blood pDC levels by 4 weeks post infection (wpi) and moderate mDC recruitment by 14 dpi. Aged animals infected with either strain showed reduced levels of pDCs in the blood and a late mDC recruitment at 4 wpi [59]. These data indicated that aged macaques have defective and delayed DC recruitment in the blood. Monocytes/macrophage numbers were increased only in adult animals, with a more dramatic increase reported in CHIKV-37997 infected animals compared to CHIKV-LR infected rhesus macaques [59]. Interestingly, beginning at 2 dpi the monocyte and mDC populations in the peripheral blood of CHIKV-LR infected rhesus macaques become highly activated demonstrating increased surface expression for CD169. The level of CD169 returns to baseline between 5–7 dpi suggesting that this level of activation is transient and reflects the level of viremia (Streblow: unpublished data [79]).

In the cynomolgus macaque model of CHIKV infection, macrophages were not only reported to traffic to lymphoid tissues, but also stained positive for CHIKV antigen. CHIKV antigen positive CD68^+^ macrophages were also reported in the spleen and lymph nodes of CHIKV-LR infected cynomolgus macaques as early as 2 dpi until 3 months after infection [53]. Further analysis of spleen CD68^+^ macrophages, utilizing *in situ* hybridization and immunohistochemistry, confirmed the presence of CHIKV genomes in macrophages at both 6 and 19 dpi [53].

##### Cytokine Response in CHIKV-infected NHP

A key component of the immune response against CHIKV is the production of cytokines and chemokines. In humans, the type 1 IFN response plays a key role in controlling the severity of CHIKV infections [22,49,80]. In the cynomolgus macaque model, IFN-α/β levels were increased 1–2 dpi and then fell sharply at 4 dpi, following the same pattern as viremia [53]. In other studies with NHPs, specifically rhesus macaques, plasma levels of bioactive IFN were measured at 3 dpi through activation of interferon stimulated gene (ISG) expression. Fibroblasts treated with plasma from adult animals increased ISG expression but not when treated with serum from aged animals [59]. This demonstrated that aged animals are defective in their ability to produce functional type 1 IFNs.

The plasma levels of numerous other proinflammatory cytokines and chemokines also correlated with levels of peak viremia in cynomolgus macaques. IFN-α/β, CCL2, IL-6, and IFNγ levels increased sharply with viremia at 2 dpi in the blood [53]. Other cytokines and chemokines that showed a significant increase over baseline levels during infection of cynomolgus macaques included TNF-α, CCL-3 and CCL-4 [53]. A similar pattern of increase in cytokines with levels of viremia was reported for pregnant rhesus macaques infected with CHIKV. Peak levels of cytokines IL-2, IL-6, IL-15, IL-1Ra, and the chemokines CCL-2 and VEGF increased at 3 dpi and then decreased to baseline following the peak and decline of viremia (Table 1) [42].

##### Antiviral T-Cells in CHIKV-Infected NHP

The exact role that T-cells play in controlling CHIKV infection is still unclear. During human infection of CHIKV CD8^+^ T-cells seem to be preferentially activated during the first few days of infection followed by a switch to CD4^+^ T-cells [22,81]. At later stages during chronic infection, the IFN-γ T-cell response appears to be driven primarily by CD8^+^ T-cells [22]. Mouse models have been key in demonstrating the importance of the adaptive immune response in controlling CHIKV infection [45]. CHIKV infection of laboratory mouse strains demonstrated the ability of both CD4^+^ and CD8^+^ T-cells to infiltrate the joints [48,82]. According to these models, CD4^+^ T-cells may have an important contributing role to the severity of inflammation and joint damage, recapitulating what is seen in autoimmune arthritis [47]. In rhesus macaques, CD4^+^ and CD8^+^ T-cells proliferate normally: central memory (CM) T-cell subsets were generally detected prior to effector memory (EM) T-cell subsets. There was a diminished T-cell response in aged animals infected with CHIKV-37997 compared to adult animals infected with the same CHIKV strain [59]. Rhesus macaques infected with CHIKV develop T cell responses specific for several CHIKV proteins as shown by IFN-γ ELISPOT analysis. The magnitude of antigen-specific T-cell responses in adult animals was higher compared to aged animals. T-cells specific for nsp1 were highest in frequency for both adult and aged animals. The breadth of T cell responses to CHIKV proteins differed in adult compared to aged animals; adults responded well to nsp1, capsid, E2, and nsp3 while aged animals responded mainly to nsp1 and nsp4 [59]. The results from these and other studies suggest that aged animals mount reduced and delayed T cell responses compared to adult animals to CHIKV infection, which may be a contributing factor to increased persistence in aged animals.

##### NHP Humoral Responses against CHIKV

Human infection with CHIKV results in rapid seroconversion. Anti-CHIKV antibodies appear within the first week of infection, but can be detected as early as the day of symptoms onset [55,58]. IgM peaks at 2–3 dpi and can persist at low levels for weeks to months. IgG anti-CHIKV antibodies are detectable 10–13 days post-onset and remain detectable for years after infection [83]. Antibodies from convalescent patients, *in vitro* are highly neutralizing, and *in* *vivo* can protect mice against CHIKV infection when given both prophylactically and therapeutically [84]. Some of the earliest examples of detecting and measuring anti-CHIKV antibodies in NHPs are from Binn *et al.* in 1967, and Nakao *et al*. in 1973 [51,60]. Binn *et al.* reported that in sera collected from Japanese monkeys (*Macaca fuscata)* infected with either the CHIKV-Asian or African strain, neutralizing antibodies were present 30 dpi as determined by plaque-inhibition assays [51]. Nakao *et al*. utilized two forms of inactivated CHIKV, UV-inactivated or formalin treatment, to generate an antibody response in inoculated Japanese monkeys [60]. Animals were given three injections of inactivated virus at 0, 2, and 9 weeks. After the second inoculation neutralizing antibodies were present (400, 50% PRNT) in the sera of animals, followed by a dramatic increase after the third injection [60]. UV-inactivated CHIKV was a far superior inducer of antibodies than the formalin treated CHIKV.

In the limited number of studies that have investigated NHP antibody response to CHIKV, the kinetics of the antibody response was similar to what has been described for humans. Anti-CHIKV antibodies in serum samples from CHIKV-LR infected cynomolgus macaques were detectable at 9 dpi. At this early time point 90% of the response was made up of the IgM isotype. By 16 dpi the IgM antibody response was replaced by IgG [78]. At later times post infection (100 and 180 dpi), IgG antibodies remained detectable (Table 1) [78].

Comparable antibody response kinetics was reported for rhesus macaques infected with CHIKV-LR and CHIKV-37997. In this model, anti-CHIKV IgG end point titers were compared in aged and adult animals using ELISA plates coated in either purified virions or cellular lysates from infected cells. For CHIKV-LR and CHIKV-37997 infected adult and aged animals, anti-CHIKV antibodies peaked near 21 dpi. The antibody kinetics were similar between the two age groups, but aged animals had significantly reduced IgG levels against virions [59]. Furthermore, when cellular lysates were used as a coating antigen, instead of purified virions, anti-CHIKV levels increased until 35 dpi, with similar levels reported for both adult and aged CHIKV-LR or CHIKV-37997 infected animals [59]. In pregnant rhesus macaques, anti-CHIKV antibodies were detectable at 7 dpi and continued to rise until 21 dpi [42]. The antibodies were highly neutralizing at 14 and 21 dpi for pregnant rhesus macaques infected with CHIKV-DHS-4263 compared to animals infected with CHIKV-37997 [42].

CHIKV-LR or CHIKV-37997 infection induces robust proliferation of memory B cell subsets that precedes the detection of IgG in adult rhesus macaques. As described for T cells, B cell proliferation in aged animals was also delayed and decreased [59]. MZ-like B-cell proliferation was detected at 10 dpi, peaked at 14 dpi, and returned to baseline by 21 dpi in adult CHIKV-LR infected rhesus macaques. Compared to adult animals, proliferation of MZ-like B-cells in aged animals was delayed. Proliferation of memory B-cells in adult rhesus-macaques infected with either CHIKV-LR or 37997 was biphasic; two peaks in proliferation occurred at 14 dpi and 28 dpi [59]. In aged animals, proliferation of memory B-cells was decreased and the second peak at 28 dpi was absent. The lack of this second peak could be due to the waning immune response caused by age [59].

Both NHPs and humans develop similar CHIKV antibody epitope recognition after CHIKV infection. The N-terminal region of the E2 glycoprotein was highly recognized by anti-CHIKV antibodies from CHIKV-LR infected cynomolgus macaques. Antibodies recognizing the same region from human patients infected with CHIKV has been found to provide a long-lasting protective response during the whole course of disease [85,86]. The specific E2 epitope recognized by human antibodies is called E2EP3 (amino acids 2800-2818) [86,87]. The same E2EP3 region is recognized by anti-CHIKV antibodies in sera of CHIKV-infected macaques up to 100 dpi [78]. There was only one other common E2 epitope identified by antibodies from both patients and cynomolgus macaques (amino acids 3025-3058) [78,85,86]. Anti-E2EP3 antibodies from infected cynomolgus macaques make up a large percentage of the antibody responses during the early stage of infection, which has also been shown for humans [78,86]. At later stages of disease, the convalescent and recovery phase, there is an equal distribution of the epitopes recognized by anti-CHIKV cynomolgus macaque antibodies including linear B-cell epitopes from E2, E3, capsid, nsp3, nsp1 and nsp4 proteins [78].

#### 2.3. Efficacy Testing of CHIKV Vaccines and Therapeutics in NHP

##### 2.3.1. CHIKV Vaccine Trials in NHP

A benefit to the NHP model is the ability to test the efficacy of vaccines and therapeutics in an outbred species while maintaining controls over experimental conditions. NHPs have been used as models to test the antibody producing potential of CHIKV treatments and vaccines [60,76]. Early studies showed that vaccination of Japanese Macaques with UV-inactivated or formalin treated CHIKV resulted in a neutralizing antibody response that could be enhanced by booster immunizations [60].

One of the early vaccines was a live-attenuated CHIKV 181/clone 25 derived from serial passaging the virus in MRC-5 cells [77]. Immunization of rhesus macaques resulted in low viremia and induced production of neutralizing antibodies by day 14. Immunized animals were protected from subsequent challenge, demonstrated by absence of virus in the serum. The vaccine progressed to phase II clinical trials but was halted because a small percentage of volunteers developed transient arthralgia [88].

Two live-attenuated CHIKV-IRES vaccine candidates, which contain a picornavirus internal ribosome entry site (IRES) to attenuate the virus, were recently tested in cynomolgus macaques [74]. A single vaccination with either CHIKV/IRES construct protected the cynomolgus macaques from viremia, fever, and rise in heart rate. At 35 dpi, virus titers were analyzed in axillary, bronchial, and inguinal lymph nodes and serum, and no virus was found. One limitation of the study was that they did not titer muscle, spleen, or liver tissues previously determined to be sites of persistence in the cynomolgus macaque model [53].

A CHIKV virus-like particle (VLP) vaccine protected rhesus macaques from CHIKV challenge [76]. Compared to control animals, VLP-vaccinated animals did not develop viremia, or show signs of acute CHIKV disease, demonstrating protection from infection. The VLP vaccine required at least two vaccinations for production of high titer neutralizing antibodies and passed phase 1 clinical trials [89]. The vaccines and therapeutics for CHIKV that have been tested in NHPs are listed in Table 2.

**Table 2:** CHIKV Vaccines and Therapeutics Tested in NHPs.

Vaccine Type	Number of Vaccinations	Immunogenicity Measured	Challenge Dose/Strain/Route	Reference
Formalin-inactivated/UV-inactivated	3	Neutralizing antibodies	Not reported	[60,90]
Live-attenuated	1	Neutralizing antibodies	10^5^ PFU / CHIKV-15561 / i.m.	[77]
Virus-Like Particle (VLP)	3	Neutralizing antibodies	10^10^ PFU / CHIKV-LR/ i.v.	[76]
DNA	5	Neutralizing antibodies / T cells	Not reported	[91]
CHIKV/IRES	1	Neutralizing antibodies	10^5^ PFU / CHIKV-LR / s.c.	[74]
**Therapeutic Type**	**Infection Dose/Strain/Route**	**Therapeutic Delivery**	**Measure of Protection**	
Antibody Therapeutic	10^7^ PFU / CHIKV-LR / s.c.	1 & 3 dpi / i.v.	Virus dissemination	[63]

##### 2.3.2. CHIKV Antiviral Therapeutic Trials in NHP

Immunotherapeutics have also been tested in rhesus macaques as prophylactic treatment options for CHIKV infections. Two humanized monoclonal antibodies (mAb CHK-152 and CHK-166) that recognize epitopes within the structural EI and E2 proteins, previously shown to be protective as a prophylactic treatment in *Ifnar^−/−^* mice [75], were tested in combination as a post-exposure therapeutic for CHIKV in rhesus macaques [63]. In this study, rhesus macaques were infected subcutaneously in both arms with a total of 10^7^ pfu CHIKV-LR distributed over 10 sites. Then at 1 and 3 dpi the animals were intravenously infused into the saphenous vein with either a cocktail of anti-CHIKV mAbs CHK-152 and CHK-166 or a control mAb (WNV E16). Although both groups had detectable CHIKV in the serum at 1 dpi, no virus was detected in serum at 2 dpi of animals that received CHIKV mAb therapy. At 7 dpi viral RNA load and tissue dissemination was quantified. Viral burden was similar in the arm muscles, joints and axillary lymph nodes between the anti-CHIKV mAb treated group and the control group. However, viral burdens in the joints and tissues of the legs, as well as the inguinal lymph node that drains the leg were reduced in the animals that received the anti-CHIKV mAb compared to controls. Measurement of viral burdens in other organs revealed that virus levels were also reduced in the kidney, lungs, and mesenteric lymph nodes in animals treated the anti-CHIKV mAb. In contrast there was no difference in the viral burden in the heart and spleen between the two study groups.

These results have generated interesting mechanisms about how CHIKV disseminates from the initial point of infection in NHPs. From this study, we speculate that CHIKV travels from the arm (the infection site) into the draining axillary lymph nodes where it then causes widespread viremia in the blood. Once in the blood the virus quickly infects the spleen and heart. Then it establishes infection in the distal joints and muscles, their draining lymph nodes as well as the secondary organs like the lungs and kidneys. However, animals that received antibody therapy were much more limited in their level of viral dissemination to the distal joints, muscles, and secondary organs. The ability to accurately detect blockage of viremia, as well as CHIKV dissemination to tissues away from the site of infection in an animal model, as was demonstrated in this study, makes rhesus macaques an ideal preclinical model for testing therapeutics [63].

## 3. Conclusions

Experimental infection of cynomolgus or rhesus macaques with CHIKV provide models that recapitulate many of the key features of human CHIKV infection. The acute stage of infection in both macaque models is characterized by the development of viremia, fever, rash, increase in cytokines and chemokines, and leukopenia. CHIKV replicates locally at the site of infection and quickly disseminates to tissues where it replicates robustly, especially spleen, muscle, and joints (Table 1). The early convalescent stage of infection is marked by a return of leukocyte numbers in the blood to normal, along with infiltration of infected macrophages along with virus into lymphoid organs and joints, as shown in the cynomolgus macaque infection model. During the convalescent stage of infection, the rhesus macaque model was key in defining the kinetics of the adaptive immune response against CHIKV. Cynomolgus macaques show viral persistence, which has been the most difficult aspect of CHIKV infection to model in small animals. Infectious virus was isolated from infected animals up to 2 months post infection in lymphoid organs and liver. In these tissues, CHIKV was detected in activated macrophages, providing further insight into the nature of CHIKV persistence [53]. The similarities between CHIKV infections of NHPs and humans make NHPs an ideal model to not only study the immune response to CHIKV, but also to evaluate the potential of anti-viral treatments and vaccines.
